# Characteristics of Methyl Cellulose Based Solid Polymer Electrolyte Inserted with Potassium Thiocyanate as K^+^ Cation Provider: Structural and Electrical Studies

**DOI:** 10.3390/ma15165579

**Published:** 2022-08-14

**Authors:** Shujahadeen B. Aziz, Elham M. A. Dannoun, Ari A. Abdalrahman, Rebar T. Abdulwahid, Sameerah I. Al-Saeedi, Mohamad A. Brza, Muaffaq M. Nofal, Ranjdar M. Abdullah, Jihad M. Hadi, Wrya O. Karim

**Affiliations:** 1 Hameed Majid Advanced Polymeric Materials Research Laboratory, Physics Department, College of Science, University of Sulaimani, Qlyasan Street, Kurdistan Regional Government, Sulaimani 46001, Iraq; 2 The Development Center for Research and Training (DCRT), University of Human Development, Sulaimani 46001, Iraq; 3 Associate Chair of the Department of Mathematics and Science, Woman Campus, Prince Sultan University, P.O. Box 66833, Riyadh 11586, Saudi Arabia; 4 Department of Physics, College of Education, University of Sulaimani, Old Campus, Sulaimani 46001, Iraq; 5 Department of Chemistry, College of Science, Princess Nourah bint Abdulrahman University, P.O. Box 84428, Riyadh 11671, Saudi Arabia; 6 Medical Physics Department, College of Medicals and Applied Science, Charmo University, Sulaimani 46023, Iraq; 7 Department of Mathematics and Science, Prince Sultan University, P.O. Box 66833, Riyadh 11586, Saudi Arabia; 8 Nursing Department, College of Nursing, University of Human Development, Kurdistan Regional Government, Sulaimani 46001, Iraq; 9 Department of Chemistry, College of Science, University of Sulaimani, Qlyasan Street, Kurdistan Regional Government, Sulaimani 46001, Iraq

**Keywords:** solid polymer electrolyte, FTIR study, impedance analysis, ion transport parameters, dielectric properties

## Abstract

The attention to a stable and ionic conductive electrolyte is driven by the limitations of liquid electrolytes, particularly evaporation and leakage, which restrain their widespread use for electrochemical device applications. Solid polymer electrolyte (SPE) is considered to be a potential alternative since it possesses high safety compared to its counterparts. However, it still suffers from low device efficiency due to an incomplete understanding of the mechanism of ion transport parameters. Here, we present a simple in situ solution casting method for the production of polymer-based electrolytes using abundantly available methylcellulose (MC) doped at different weight percentages of potassium thiocyanate (KSCN) salt. Fourier transform infrared (FTIR), and electrochemical impedance spectroscopy (EIS) methods were used to characterize the prepared samples. Based on EIS simulation and FTIR deconvolution associated with the SCN anion peak, various ion transport parameters were determined. The host MC medium and KSCN salt have a strong interaction, which was evident from both peak shifting and intensity alteration of FTIR spectra. From the EIS modeling, desired electric circuits correlated with ion movement and chain polarization were drawn. The highest ionic conductivity of 1.54 × 10^−7^ S cm^−1^ is determined from the fitted EIS curve for the film doped with 30 wt.% of KSCN salt. From the FTIR deconvoluted peak, free ions, ions in contact with one another, and ion aggregates were separated. The extracted ion transport parameters from the EIS method and FTIR spectra of the SCN anion band confirm that both increased carrier concentration and their mobility were crucial in improving the overall conductivity of the electrolyte. The dielectric investigations were further used to understand the conductivity of the films. High dielectric constants were observed at low frequencies for all MC:KSCN systems. The dispersion with a high dielectric constant in the low-frequency band is ascribed to the dielectric polarization. The wide shift of *M*″ peak towards the high frequency was evidenced by the MC-based electrolyte impregnated with 30 wt.% of KSCN salt, revealing the improved ionic movement assisted with chain segmental motion. The AC conductivity pattern was influenced by salt concentration.

## 1. Introduction

Recently, the design of emerging clean and renewable energy, and its related technology breakthroughs have gained interest due to the widespread use of fossil fuel, the restrictions of a global society on carbon dioxide emissions, and the cost of crude oil. The development of lithium metal-based devices has attracted a lot of attention, signifying their enormous potential as power sources. However, despite its high-energy density and wide range of applications, there are still many problems that impact its performance, rate capability, safety, and cost [1,2,3]. 

Many attempts have been made to utilize benign and harmless electrolytes in energy storage devices. However, it is hard to achieve satisfactory devices with suitable sizes and shapes that compete with liquid electrolytes [3,4]. Therefore, researchers have to think about safe, efficient, and relatively stable solid polymer electrolytes (SPEs). SPEs have several properties, including lightweight, satisfactory thermal stability, high flexibility, low cost, ease of handling, and harmlessness [5,6]. A dye-sensitized solar cell, a battery, a supercapacitor, an electrochemical double-layer capacitor (EDLC), and a fuel cell are just a few examples of electrochemical devices in which the cells typically consist of an electrolyte and two electrodes. The electrolyte plays at the heart of the cells in the electrochemical devices [7,8,9,10,11,12]. Polymer electrolytes (PEs) have been the subject of intensive research in an effort to develop systems with good thermal, electrical, electrochemical, and mechanical properties that are also inexpensive and environmentally friendly [7,13,14]. However, the SPEs are not free from drawbacks, the most common one is the low ionic conductivity [15].

Global warming and water contamination are two of the negative effects of discharging plastic garbage into the environment. These two problems raise the awareness and motivation of researchers to investigate biodegradable and biocompatible polymers [16,17]. Natural polymers are attracting the attention of researchers due to their abundance, cost-effectiveness, biodegradability, and biocompatibility [18,19]. In other words, green or natural biopolymers degrade gradually in contrast to synthetic or human-made polymers [20,21]. As a host natural polymer, SPE-based polymers have been investigated previously, such as chitosan [22], starch [23], and poly(vinyl alcohol) (PVA) [24]. Excellent chemical, physical, and relative ionic conductivity combine to produce these natural polymers’ remarkable performance [25]. Additionally, natural polymers are renewable, inexpensive, abundant, nontoxic, biodegradable, and biocompatible [26,27]. 

Natural polymers such as cellulose in their neat state are insoluble in water [28,29]. Thus, modification of cellulose is necessary to solubilize it in aqueous media. Herein, the incorporation of methyl chloride into the cellulose (methylation) results in the formation of methyl cellulose (MC). This modification of cellulose by methylation is not expensive and eco-friendly, in addition to its ability to form a film. Furthermore, MC has transparency as well as convincing mechanical and electrical properties [30,31]. 

Cations usually interact with oxygen bound within the backbone of MC via a dative bond. MC has many functional groups rich in lone pair electrons, including C-O-C, O-H, and O-CH_3_, which are responsible for the conduction of ions [32]. The MC is well-known as an amorphous polymer, possessing T_g_, in the range of 184–200 °C [33]. MC was chosen as the host polymer in this study because its properties change when KSCN is added at different concentrations. In addition, the system becomes an ionic conducting phase beyond dissolving salts in a high molecular weight host matrix. From an atomic level perspective, the local relaxation in high molecular weight polymer provides degrees of freedom as such in liquids, in addition to cordial. Furthermore, the mentioned polymer system also provides a compatible interface with electrode materials [34].

The σ_dc_ of 9.334 × 10^−5^ S/cm was recorded by Mallaiah et al. for the PEO:PVdF:NaNO_3_ system [35]. Moreover, the σ_dc_ of 6.34 × 10^−7^ S/cm has been documented for blended CS with starch doped with NH_4_NO_3_ SPEs [36]. Importantly, insertion of 10 wt.% of AgTf salt into CS with recording 4.2 × 10^−8^ S/cm ionic conductivity [37]. The MC:PVA system has an ionic conductivity of 1.53 × 10^−5^ S/cm when 50% of sodium iodide (NaI) is loaded into it [38]. Despite the host polymer enriching in functional groups, the absence of free ion carriers results in weak conduction. Thus, choosing a suitable dopant salt for the polymeric host medium is decisive. Several characteristics, such as lattice energy of the salt, cation size, ion concentration, and mobility have to be taken into consideration in enhancing the ionic conductivity of the SPE [39,40,41,42]. While lithium-based salts have a higher lattice energy than potassium thiocyanate (KSCN) salts (616 kJ/mol); K-salts are safer [43,44]. The dielectric constant of the polymer host and the rate at which ions aggregate appear to be important determinants of ionic conduction as a parameter for evaluating the effectiveness of an SPE [45,46]. Thus, to fully understand how ions interact with polymers between molecules and how ions move through SPE, it is important to look at the dielectric properties of the host polymer [47,48].

An SPE based on MC polymer with various amounts of KSCN dopant salt will be synthesized using the solution casting process, which is being used in this study. Analysis of the blended polymer was carried out utilizing FTIR and electrochemical impedance spectroscopy (EIS). Free ion concentration and its effect on ion conductivity are hotly debated issues. Additional characterization and analysis are carried out to better understand how salt’s ionic charge carriers interact with the polymer’s polar functional groups. For example, deconvoluted FTIR was utilized to categorize aggregated and free ions inside the polymer host. Using EIS and EEC modeling, the circuit architecture for each MC electrolyte will be shown. Then, it will be used to figure out the conductivity of ions by measuring the bulk resistance in circuit designs.

## 2. Materials and Methods

### 2.1. SPE Preparation

MC polymer and KSCN were purchased from Sigma-Aldrich and used as received without purification. The solution cast process was used to insert a range of quantities of KSCN into the MC polymer matrix. The first solution was made by dissolving 1 g of MC in 100 mL of 1% acetic acid and stirring with a magnetic stirrer until a homogeneous aqueous solution was achieved. The SPEs are prepared by adding various amounts of KSCN salt into the MC solution separately and stirred for 24 h to gain a fully dissolved salt and homogenous solution. The samples were coded as MCK1, MCK2, MCK3, MCK4, and MCK5 for the inclusion of 10, 20, 30, 40, and 50 wt.% of KSCN salt. Afterward, the samples were cast into dry, clean, labeled Petri dishes and left for 14 days for the films to form at ambient temperature with 20% relative humidity. To ensure the complete dryness of the films, a further drying process was carried out by putting the samples in a desiccator filled with silica gel. Ultimately, a uniform solvent free of ~0.026 cm thick SPEs films was achieved and ready to be characterized. 

### 2.2. Electrical Impedance Spectroscopy (EIS)

**Figure 1:**
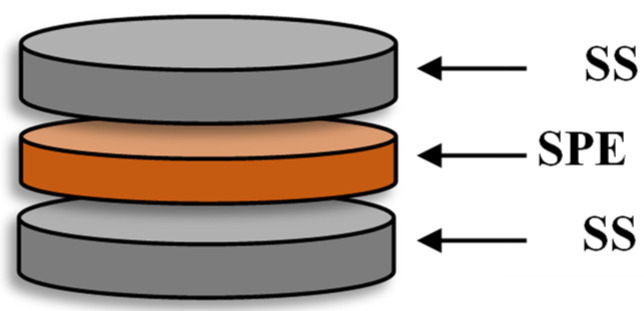
Schematic representation of the cell used to measure conductivity.

Solid-state materials can be examined by utilizing complex impedance spectroscopy (CIS) in order to better understand their electrochemical properties [49]. Materials’ electrical characteristics and their relationship to electrodes with electronic conductivity are better understood using CIS. The SPE films were prepared by cutting them into tiny discs (1 cm in radius), in which a spring was used to compress the electrolyte between two stainless steel electrodes. The HIOKI 3531 Z Hi-tester employed for the impedance to be achieved. The room temperature and from 100 Hz to 2 MHz the frequency ranged when the device is linked to the computer. In order to obtain the real and imaginary components of impedance, we used software to control all measurements and computations. The bulk resistance was determined by plotting the actual impedance axis with the intercept of the plot. The equation for calculating conductivity is presented below [50]:

(1) $$\sigma_{DC}=\left[\frac{1}{R_{b}}\right]\times \left[\frac{t}{A}\right]$$

where, *A* is the film’s area, whereas *t* is its thickness. The measurements of EIS were made from a cell consisting of stainless steel SS |SPE film| SS as explained in Figure 1.

The real and imaginary components of permittivity ($\varepsilon^{*}$) and modulus ($M^{*}$) can be calculated from the complex impedance ($Z^{*}$) using the following equations [51,52,53].

(2) $$Z^{*}=Z^{\prime }-jZ^{\prime \prime }$$

(3) $$\varepsilon^{*}=\varepsilon^{\prime }-j\varepsilon^{\prime \prime }=\frac{1}{j\omega \varepsilon_{o}Z^{*}}$$

(4) $$M^{*}=\frac{1}{\varepsilon^{*}}=j\omega C_{o}Z^{*}=M^{\prime }+jM^{\prime \prime }$$

From Equations (2)–(4) the following relationships can be achieved,

(5) $$\varepsilon^{\prime }=\frac{Z^{\prime \prime }}{C_{o}\omega \left(Z^{\prime 2}+Z^{\prime \prime 2}\right)}$$

(6) $$\varepsilon^{\prime \prime }=\frac{Z^{\prime }}{C_{o}\omega \left(Z^{\prime 2}+Z^{\prime \prime 2}\right)}$$

(7) $$M^{\prime }=\frac{\varepsilon^{\prime }}{\left(\varepsilon^{\prime 2}+\varepsilon^{\prime \prime 2}\right)}=C_{o}\omega \;Z^{\prime \prime }$$

(8) $$M^{\prime \prime }=\frac{\varepsilon^{\prime \prime }}{\left(\varepsilon^{\prime 2}+\varepsilon^{\prime \prime 2}\right)}=C_{o}\omega Z^{\prime }$$

where $\varepsilon^{\prime }$, and $\varepsilon^{\prime \prime }$ as usual are the dielectric constant, and dielectric loss, *C_o_* is the vacuum capacitance ($\varepsilon_{o}$ A/t). The real part and imaginary part of the complex electric modulus are denoted by *M*′ and *M*″, respectively. The angular frequency *ω* is equal to (2*πf*), with *f* representing applied field frequency.

### 2.3. Fourier Transform Infrared (FTIR) Spectroscopy

In this study, the complexation between the components of the SPE systems was confirmed using FTIR spectroscopy. FTIR spectra of the films were acquired using a Perkin Elmer Spotlight 400 spectrometer (Waltham, MA, USA) in the wavenumber range (400–4000 cm^−1^) at a resolution of 1 cm^−1^. Both the Gaussian–Lorentzian function and the deconvolution method were employed to extract the overlapping peaks and fit the curves from which ion transport parameters were determined. 

## 3. Results and Discussion

### 3.1. Impedance Analysis

**Figure 2:**
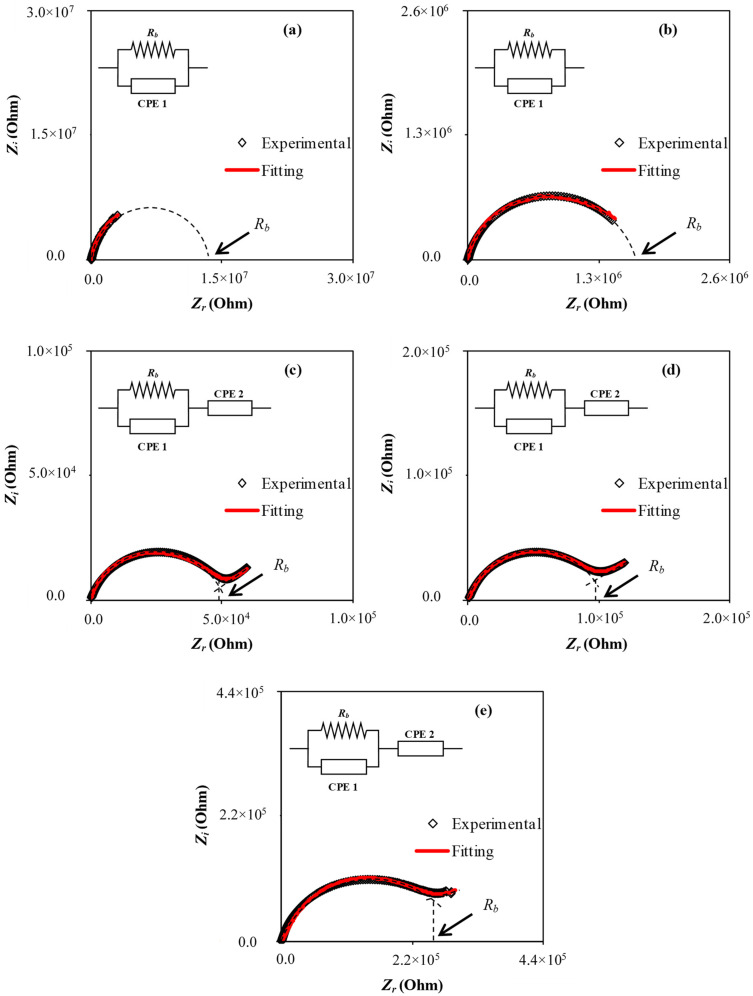
The EIS and simulated data for (**a**) MCK1, (**b**) MCK2, (**c**) MCK3, (**d**) MCK4, and (**e**) MCK5.

Ion dynamics in PE systems can be well characterized using impedance spectroscopy. The outcomes of the impedance of the present SPE were used to have a comprehensive understanding of the electrolyte conductivity and its frequency behavior. Both MCK1- and MCK2-based polymer electrolytes have the Cole-Cole plots (Nyquist plots) at room temperature as exhibited in Figure 2a,b in which a semicircle is seen only. A high-frequency semi-circle and a low-frequency spike are frequently observed when the salt concentration is high Figure 2c,d. Bulk resistance (resulting from ion mobility) and bulk capacitance originating from immobile polymer chains are combined to generate a high-frequency semi-circle [51]. The size of the high-frequency semi-circle decreased considerably as KSCN concentrations climbed. The emergence of a spike from 30 wt.% to 50 wt.% of KSCN demonstrates an increase in conductivity [54,55]. The spike length is reduced at 40 and 50 wt.% of KSCN salt, indicating a reduction in conductivity. Calculating bulk resistance (*R_b_*) at low and high salt concentrations is made easy using the impedance plot’s inset. It is fascinating to see that when KSCN concentration increases up to 40 wt.%, *R_b_* declines.

Impedance spectroscopy analysis following EECs modeling is a simple, fast, and comprehensive method for obtaining a full view of the electrolyte system [56]. The picture of the measured impedance plots in relation to the equivalent circuit is presented in the insets of Figure 2, which includes *R_b_* for the sample’s charge carriers and two CPEs (constant phase elements). *R_b_* and CPE1 make up the high-frequency zone, while CPE2, which is derived from the region between the electrodes and SPE that created double layer capacitance, makes up the low-frequency area. As previously mentioned, in an analogous circuit, a CPE shortened word is used instead of an ideal capacitor in the actual system. When it comes to a pure semicircular pattern, the real SPE behaves differently from an ideal capacitor [57]. The CPE has been used instead of a capacitor to represent the depressed semicircle [53]. *Z_CPE_*_1_ is the capacitance response at the electrode–solid PE interface. [57]. It is possible to write *Z_total_* as *R_b_* + *Z_CPE_* impedance as [39,58,59,60]:

(9) $$Z_{total}=R_{b}+\frac{1}{C\omega^{p}}\left[\cos\left(\frac{\pi p}{2}\right)-i\sin\left(\frac{\pi p}{2}\right)\right]$$

In the right-hand side model, there is a second term *Z_CPE_* known as the impedance of the constant phase element. Here, the reciprocal of capacitance is *K*, and the deviation of the inclined line from the real axis is represented by *p*. If *p* has the value of unity, $Z_{total}=R-\frac{j}{\omega C}\;$. Whereas, when *p* is zero, a perfect resistor is considered instead of the constant phase element, in which *Z_total_* becomes independent on frequency. Furthermore, when *p* value lies between zero and one, *CPE* behaves as an intermediate between a resistor and a capacitor, while at *p* = 0.5, the impedance is the Warburg impedance.

*Z_r_* and *Z_i_* are the real and imaginary complex impedance (Z*) values in the analogous circuit, respectively, and their mathematical basis is shown below in Figure 2c–e [39,52,59,60]:

(10) $$Z_{r}=\frac{R_{b}C_{1}\omega^{p1}\cos\left(\frac{\pi p_{1}}{2}\right)+R_{b}}{2R_{b}C_{1}\omega^{p}\cos\left(\frac{\pi p}{2}\right)+{R_{b}}^{2}C^{2}\omega^{2p}+1}+\frac{\cos\left(\frac{\pi p_{2}}{2}\right)}{C_{2}\omega^{p2}}$$

(11) $$Z_{i}=\frac{R_{b}C_{1}\omega^{p1}\sin\left(\frac{\pi p_{1}}{2}\right)}{2R_{b}C_{1}\omega^{p}\cos\left(\frac{\pi p}{2}\right)+{R_{b}}^{2}C^{2}\omega^{2p}+1}+\frac{\sin\left(\frac{\pi p_{2}}{2}\right)}{C_{2}\omega^{p2}}$$

While for Figure 2a,b, the mathematical equations are:

(12) $$Z_{r}=\frac{R_{b}C_{1}\omega^{p1}\cos\left(\frac{\pi p_{1}}{2}\right)+R_{b}}{2R_{b}C_{1}\omega^{p}\cos\left(\frac{\pi p}{2}\right)+{R_{b}}^{2}C^{2}\omega^{2p}+1}$$

(13) $$Z_{i}=\frac{R_{b}C_{1}\omega^{p1}\sin\left(\frac{\pi p_{1}}{2}\right)}{2R_{b}C_{1}\omega^{p}\cos\left(\frac{\pi p}{2}\right)+{R_{b}}^{2}C^{2}\omega^{2p}+1}$$

Figure 2 shows a good simulation (red solid lines) of the experimental impedance plots based on Equations (10)–(13) and EECs are shown in the figure insets. One can determine the ionic conductivity (σ) of SPEs samples by using Equation (1). Table 1 shows the bulk resistance and circuit elements for MC: KSCN SPE films. Table 1 reveals that as KSCN concentration is increased, higher values of DC conductivity for SPE films were recorded accordingly. Due to an increase in the number of mobile charge carriers, there is a significant decrease in $R_{b}$ with rising KSCN salt. Moreover, the charge carrier concentration increasing leads to increasing DC conductivity at ambient temperature as mathematically shown in Equation (14) [37,47,61],

(14) $$\sigma =\sum_{i}n_{i}\;z_{i}\;\mu_{i}$$

**Figure 3:**
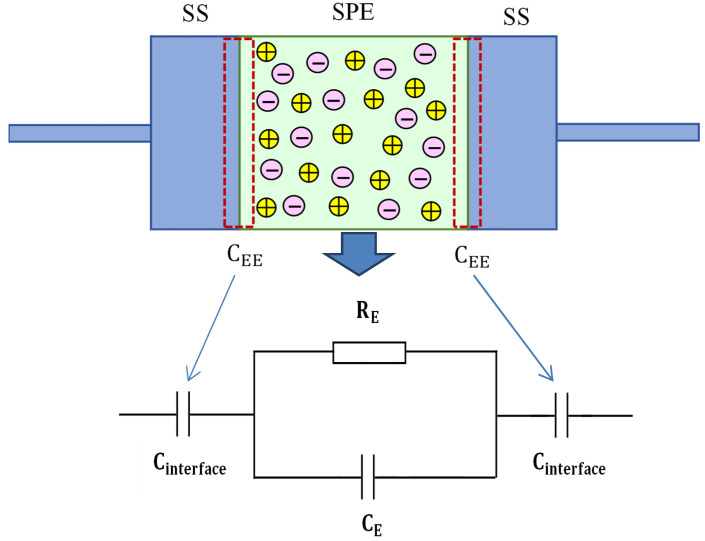
An EEC diagram for impedance graphs shows a high-frequency semicircle and a low-frequency tail as a schematic representation.

Here, *n_i_* is the density of charge carriers with the ion mobility denoted by *µ_i_*, and 1.6 × 10^−19^ C is the value of *q*. Variables such as ionic conducting charge species concentration, temperature, and carrier mobility can all affect ionic DC conductivity [62]. Thus, substantial growth in DC conductivity for the sample with 30 wt.% of KSCNis correlated to the increase in charge carrier concentration as a consequence of salt involvement at room temperature [37,47,63]. For more clarification, an EEC diagram for impedance plots, which indicates a high-frequency semicircle and a low-frequency spike as a schematic representation, is shown in Figure 3.

As the (MCKN3, MCKN4, and MCKN5) films contain a spike and a semicircle, the diffusion coefficient (*D*), mobility of ions (*μ*), and number density (*n*) of ions are determined by the below relations [63]: 

The *D* of the (MCKN3, MCKN4, and MCKN5) samples is achieved using Equation (15),

(15) $$D=\left(\frac{{\left(K_{2}\varepsilon_{o}\varepsilon_{r}A\right)}^{2}}{\tau_{2}}\right)$$

where *τ*_2_ denotes the reciprocal of angular frequency corresponding to the minimum in *Z_i_*. 

The *µ* of the above films is achieved using the Equation (16),

(16) $$\mu =\left(\frac{eD}{K_{b}T}\right)$$

where *T* stands for the absolute temperature and *K_b_* stands for the Boltzmann constant. 

Since conductivity is written by

(17) $$\sigma_{Dc}=ne\mu$$

So, the *n* of the above films is achieved by Equation (18):

(18) $$n=\left(\frac{\sigma_{dc}K_{b}T\tau_{2}}{{\left(eK_{2}\varepsilon_{o}\varepsilon_{r}A\right)}^{2}}\right)$$

Table 2 lists the parameters of ion transport for the samples.

In Table 2, the *D*, *μ*, and *n* are increased when the salt is increased. This increase in the *D*, *μ*, and also *n* increased the conductivity. The number of ions increases as the concentration of salt increases [63]. 

### 3.2. FTIR Study

**Figure 4:**
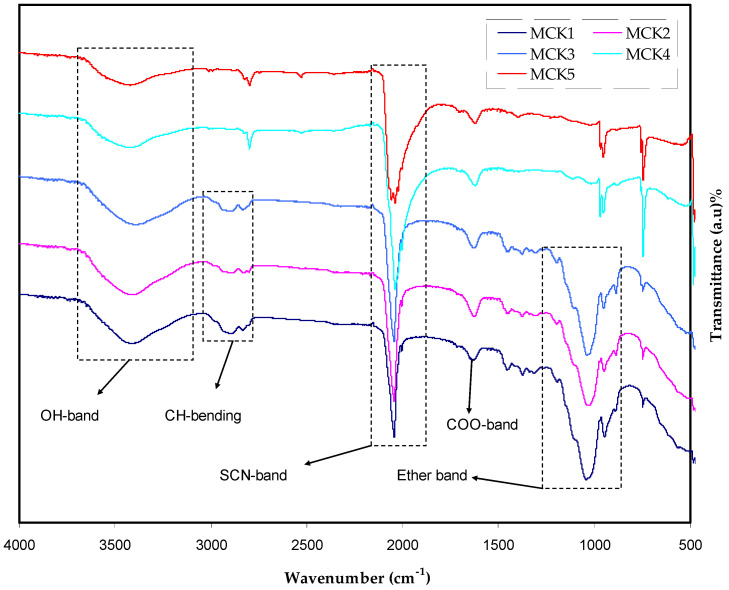
Full range of FTIR spectra for MC:KSCN systems.

Figure 4 shows the FTIR spectra at a wave number from 400 to 4000 cm^−1^ for the MC:KSCN based biopolymer electrolytes. FTIR is the best method for determining the structure and content of novel organic compounds generated during chemical processes. Position shifting and intensity fluctuation of bands in electrolyte samples are considered the best indicators of the existence of specific interaction among salts cation and polymers functional groups. Heteroatoms (such O and N) are shown to be important elements in the electrolyte and lone pair electron interaction in the polymer host [64]. It is possible to highlight SPEs system interactions between MC and KSCN by taking FTIR spectra of them. 

The existence of the C-H stretching modes seen in Figure 4 is indicated by the formation of a significant peak at roughly 2900 cm^−1^ [65,66,67,68,69], and the salt content also reduced its intensity. Furthermore, doping has been shown to have a major impact on this process, as seen in Figure 4, which depicts a large peak centered at 3359 cm^−1^ as the source of the -OH stretching [65]. As a result of both peak shifting and intensity change, a significant interaction arises between the host medium and KSCN salt. As a result of past research [33,69], this polymer has distinct vibrational frequencies in the FTIR spectra of O=C-NHR and -OH. Increased KSCN concentration causes the peak intensity to decrease and shift somewhat. In fact, the complexation process is proven by the fact that the polymer body and the KSCN salt are in close contact with each other through a coordination link.

This technique is also effective for measuring the dissociation of ions [70]. Thiocyanate anion (SCN-) has two possible reactive sites and regarded as a linear molecular ion. This ion can form bridge complexes between N and S atoms in addition to S-bonding (CS stretching) and N-bonding (CN stretching). It has been suggested by Woo et al. that the wavelengths at which the bands appear correspond to free ions at 2040 cm^−1^, pair contact ions at 2058 cm^−1^, and ions aggregates at 2074 cm^−1^ [71]. Equation (19) is used to determine the percentage of ions [63]. Ion association and dissociation were distinguished by deconvoluting the overlapped complex spectra between 2030 and 2090 cm^−1^.

When compared to other methods, such as impedance spectroscopy and Trukhan, Rice and Roth, Schutt and Gerdes models, the precision of FTIR-extracted ion transport parameters reveals that this method is superior [38,72]. FTIR spectroscopy is very sensitive to even small structural changes, making it a dependable method of spectroscopic analysis. In particular, the location and strength of some band peaks [73] clearly demonstrate this. This strategy of fitting the curve and separating single fine peaks corresponds to free ions, contact ion couples (K+. SCN-), and ion aggregates through the Gaussian–Lorentzian fitting approach and baseline correction [7,74]. Free SCN-linear anion has an estimated peak band position of 2040 cm^−1^; ion contact, and ion aggregates have peak band values of 2058 cm^−1^ and 2074 cm^−1^, respectively [71,75]. After the fitting procedure, the following equation [7,76] may be used to calculate the proportion of these ionic species in the region beneath each of the bands:

(19) $$\mathrm{percentage}\;\mathrm{of}\;\mathrm{free}\;\mathrm{ions}\;\left(\%\right)=\frac{A_{f}}{A_{f}+A_{c}+A_{a}}\times 100\%\;\;\;$$

**Figure 5:**
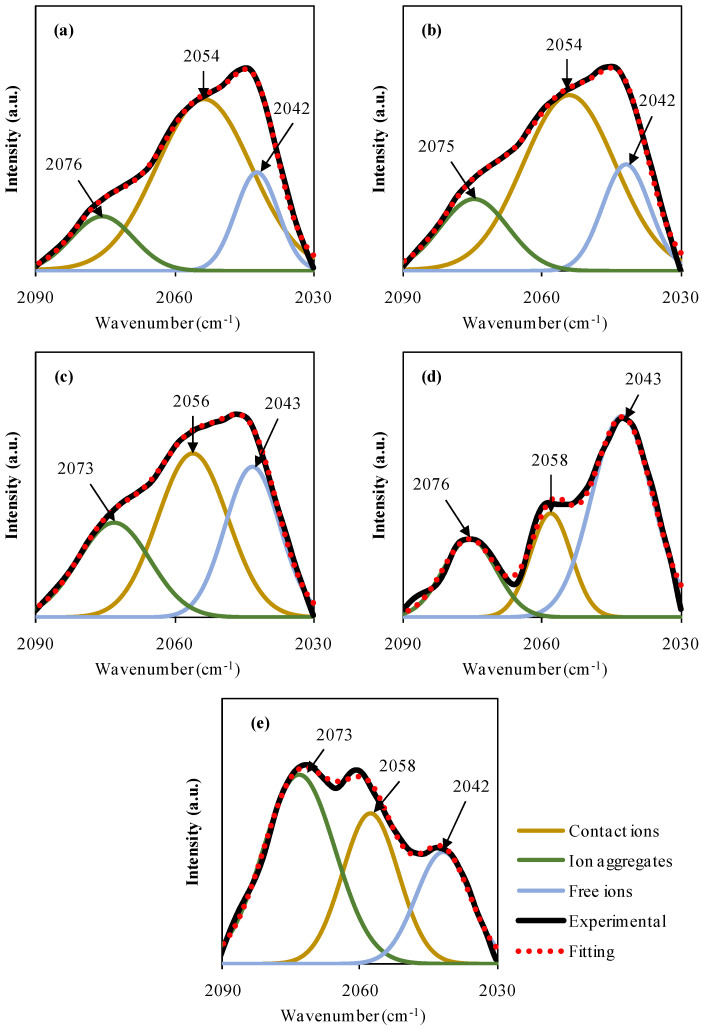
The SCN deconvoluted band for (**a**) MCK1, (**b**) MCK2, (**c**) MCK3, (**d**) MCK4 and (**e**) MCK5.

Areas under free ions, ions in contact with one another, and ion aggregates are all represented by, $A_{f}$, $\;A_{c}$, and $A_{a}$. Figure 5d shows that the CSPSK4 sample has reached its maximum salt concentration (the area of free ions grows). Increasing KSCN salt, up to 40 weight percent, causes the peak area of contact ions to steadily decrease (see Figure 5a–d). Accordingly, the number of mobile carriers is reduced as more free ions are available, which is known to have an effect on the conductivity of ions. Carboxyl methylcellulose hosts doped with NH_4_SCN [7] and oleic acid [77] have also shown similar results. The overall performance of the SPE and its ionic conduction is influenced by a number of factors, including free ions. Mobility (μ), diffusion coefficient (*D*), and the density of the carriers (*n*) are other important considerations which can also be determined using the FTIR method. The following formulae [74,76] were used to determine these parameters:

(20) $$n=\frac{M\times N_{A}}{V_{Total}}\times \left(\mathrm{free}\;\mathrm{ion}\;\%\right)$$

(21) $$\mu =\frac{\sigma }{n\;e\;}\;$$

(22) $$\;D=\frac{\mu \;K_{b}T}{e}$$

here, the total volume of the SPE is ($V_{Total}$) and (M) is the number of mole. ($N_{A}$) and (e) possess usual meanings which are $6.02\times 10^{23}{\;\mathrm{mol}}^{-1}$ and $1.6\times 10^{-19}\;C$, respectively. Both $K_{b}$ ($1.38\times 10^{-23\;}{J\;K}^{-1}$) and *T* have normal meanings. 

**Figure 6:**
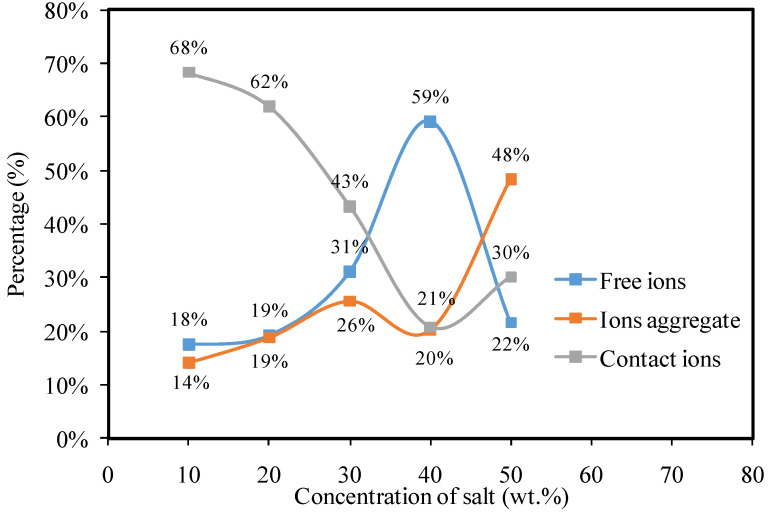
The percentage (%) of free ions, contact ions and ion aggregates vs. salt concentrations.

According to the μ and *D* values in Table 3, the optimal quantity of salt for both transport parameters is 40 wt.% of salt. Free SCN-nucleophile anion possesses two highly reactive sites for N and S bonding, in addition to a bridge complex with the host polymer (SCN bending) [78,79]. When the polymers lose their structural order and intermolecular interaction, resulting in a more amorphous phase that facilitates better ion diffusion [80]. On the other hand, at a salt concentration of 50 wt.%, both *μ* and *D* values decrease, which is consistent with the obstruction effect of free ion movement caused by the creation of an ion cloud and an ion cluster [76,77]. For more clarification, the percentage (%) of free ions, contact ions, and ion aggregates versus salt concentrations are shown in Figure 6. 

### 3.3. Dielectric Properties

#### 3.3.1. Complex Permittivity

**Figure 7:**
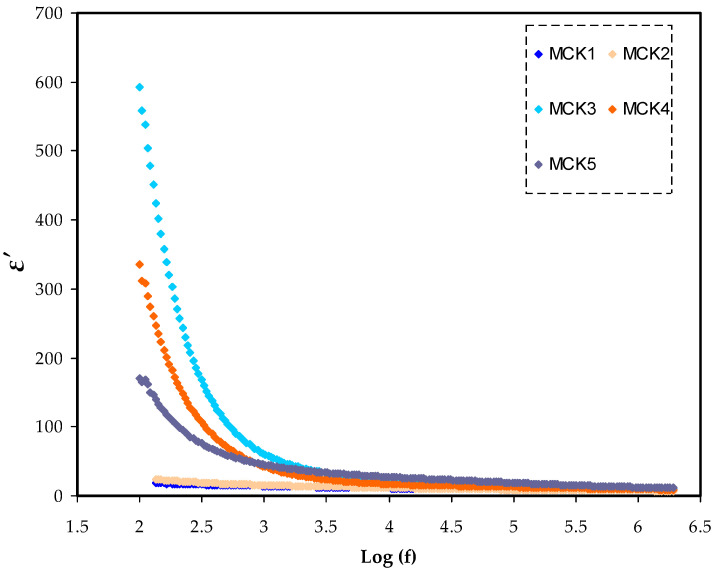
Illustrates the frequency dependence of the dielectric constant for all MC:KSN systems in frequency range (100–2 MHZ).

**Figure 8:**
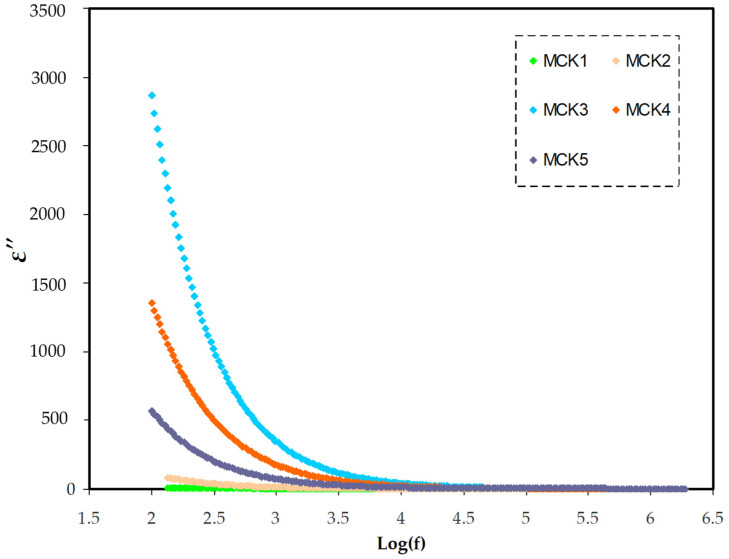
The relationship between dielectric loss versus frequency for all MC:KSN systems in frequency range (100–2 MHZ).

Through dielectric spectroscopy, the frequency-dependent dielectric properties of the medium are established. It means that the dielectric parameters can be calculated from the relationship between the measured impedance parameters versus frequency. Using a broad variety of frequencies, this approach includes the interaction of an electric dipole with an external field. The acquired data may also be used to estimate the material’s ac conductivity over a certain frequency range. The necessary energy for dipole alignment is represented by the imaginary portion ($\varepsilon^{\prime \prime }$), whereas the real part ($\varepsilon^{\prime }$) is connected to ion storage effectiveness or polarizing ability. The dielectric constant and loss were measured using Equations (5) and (6). In both dielectric constant and loss (Figure 7 and Figure 8), dispersion is seen at low frequencies. Space charge polarization was observed to exist at the electrode–electrolyte interface, as dielectric constant and loss were significant in the area [81,82,83,84,85]. It should be noted that the system with a 30 wt.% KSCN integration has the largest dielectric constant at low-frequency range. It could be caused by space charge effects in addition to electrode polarization. This suggests the presence of many charge carriers, leading to relatively high conductivity [85,86,87]. In the low frequency range, dipoles and charge carriers have sufficient time to align themselves with respect to the direction of the applied field. Electrode polarization is caused by charge buildup at the electrode/electrolyte contact, which suppresses high frequency dielectric characteristics (bulk property) [86,87,88]. The dielectric loss value is clearly greater than the dielectric constant value, showing a contribution to the dielectric loss values from carrier motion (DC conductivity) [89,90]. Due to the marginalization of the electrode–electrolyte interfaces with increasing frequency, the dielectric values are steady in high-frequency areas. The electrolyte films exhibit non-Debye behavior because the values of both ($\varepsilon^{\prime \prime }$) and ($\varepsilon^{\prime }$) drop with increasing frequency [91,92].

#### 3.3.2. Complex Electric Modulus

**Figure 9:**
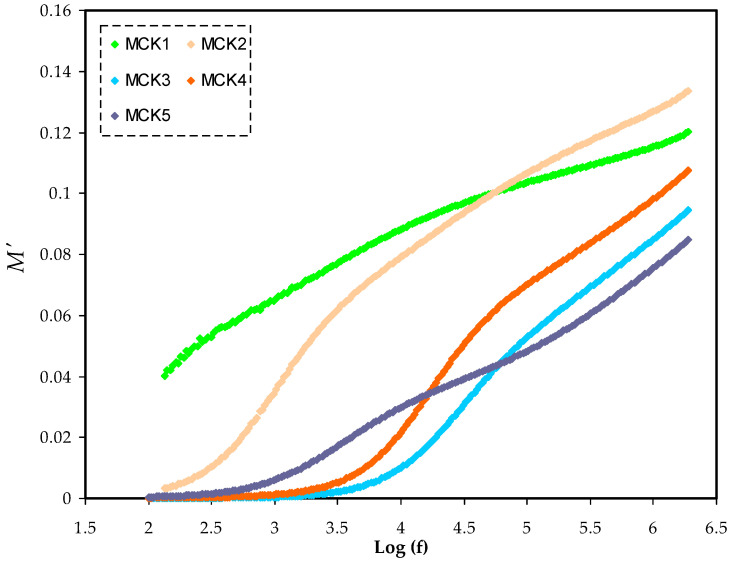
Illustrates the frequency dependence of the *M*′ for all MC:KSN systems in frequency range (100–2 MHZ).

**Figure 10:**
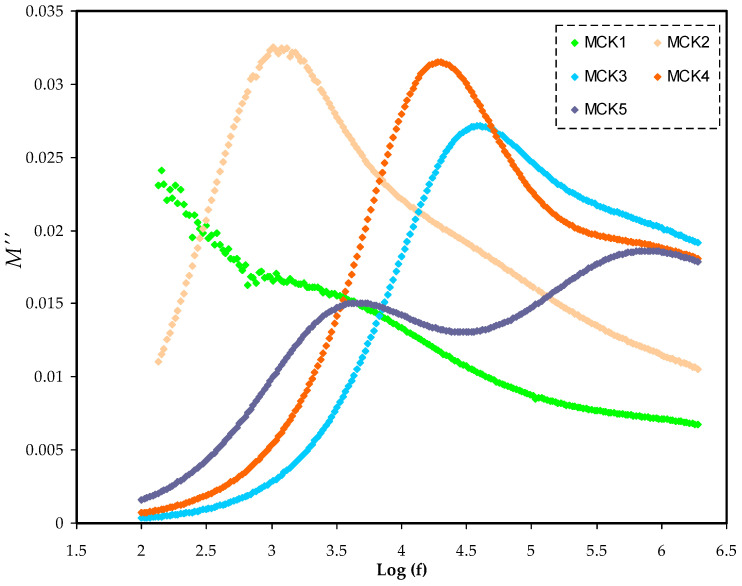
Illustrates the frequency dependence of the *M*″ for all MC:KSN systems in frequency range (100–2 MHZ).

The complex electric modulus ($M^{*}$) has the following mathematical foundation for the real (*M*′) and imaginary (*M*″) portions as shown in Equations (7) and (8). Figure 9 and Figure 10 show *M*′ and *M*″ in opposition to frequency for the PCEs at room temperature. At low frequencies, both *M*′ and *M*″ decrease as the tails lengthen, demonstrating that electrode polarization has a minor role. It implies that the polarization of the electrodes causes the SS electrodes to build up charges [93]. The spectra of *M*′ and *M*″ vary from those of $\varepsilon^{\prime }$ and $\varepsilon^{\prime \prime }$. Figure 7 and Figure 8 show the large $\varepsilon^{\prime }$ and $\varepsilon^{\prime \prime }$ values at low frequencies. In reality, the inverses of $\varepsilon^{\prime }$ and $\varepsilon^{\prime \prime }$ in $\varepsilon^{*}$ were used to create the *M*′ and *M*″ in $M^{*}$. When it comes to low-frequency capacitive behavior, these are the parameters that are most important.

Figure 9 and Figure 10 show the long tails at low frequencies. If the electrode/PE films had a considerable capacitance at low frequencies, the electrochemical double layer at the electrodes would be suppressed. Due to the fact that $\varepsilon^{\prime }$ decreases to its lowest value, *M*′ rises to its greatest value at high frequencies [86]. The *M*″ spectra of the electrolyte show a notable peak as a consequence of the relaxation of conductivity (see Figure 10). It is because *M*′ in the $M^{*}$ corresponds to $\varepsilon^{\prime }$ in the $\varepsilon^{*}$ that the loss peaks do not appear in the *M*′ spectra (see Figure 9). *M*′ is a symbol denoting the energy storage capacity of the substance [94].

### 3.4. AC Conductivity

**Figure 11:**
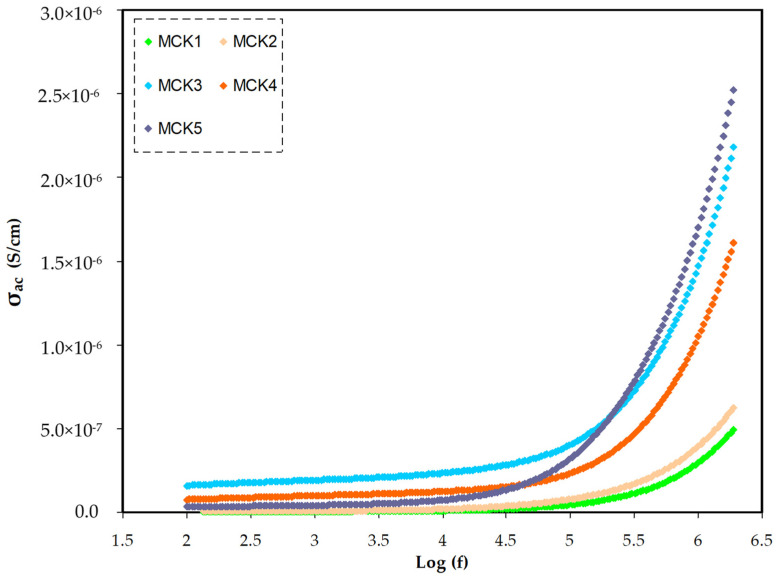
AC conductivity spectra for all MC:KSN systems in frequency range (100–2 MHZ). Based on AC spectra also MCK3 is the highest conducting electrolyte.

To realize the ion dynamics in polymer electrolytes, the frequency dependence of AC conductivity at room temperatures was examined for all samples, which can be seen in Figure 11. According to documents recorded in the literature [95] conductivity in polymer electrolytes progresses via two main mechanisms. The first mechanism is in action by ion charge migration across coordinated sites in the host polymer and results in DC contribution. The second one is in progress after increasing conductivity as a result of polymer segmental motion and polarization and results in AC dispersion. Equation (23) is applied to gain the AC conductivities:

(23) $$\sigma_{ac}=\left[\frac{Z^{\prime }}{Z^{\prime 2}+Z^{\prime \prime 2}}\right]\times \frac{t}{A}$$

In Figure 11, three different areas are recognized; firstly, the low-frequency range inclined line, resulting from the electrode polarization; secondly, the plateau area at the medium frequency, which is caused by DC conductivity at the bulk; lastly, the area of the high frequency, which shifts in position to the higher frequency as salt concentration is increased due to conductivity relaxation [96]. In the previous document, the AC conductivity dependency on the applied electrical signal frequency was applied as a method to accurately evaluate DC conductivity [97]. The extension of the plateau area to the *y*-axis is useful in estimating DC electrical conductivity. It is important to note that the rise in AC conductivity (the frequency dependence) would still be observed at relatively high frequencies. Furthermore, the rise in AC conductivity with frequency implies the presence of a hopping conduction mechanism, which enhances charge carrier hopping among localized states [98]. The origin of this strong relationship between frequency and conductivity belongs to Jonscher [99], in which the relaxation process is caused by mobile charge carriers (related to the jump relaxation model) [100]. As stated, the AC conductivity at high frequencies can be linked to the possibility of connected forward–backward hopping in conjunction with ion relaxation in the bulk of the materials. The Jonscher’s relation accurately establish the relationship between AC conductivity and charge carrier motion [101],

(24) $$\sigma_{ac}\left(\omega \right)=\sigma_{DC}+A\;\omega^{s}\left(0<s<1\right)$$

where *s* is an exponent that expresses charge carrier interactions throughout hopping processes in DC conductivity [102]. Secondly, dipole polarization (limited movement) and charge buildup at the interface result in the polarization (limited movement) component (permanent/induced). As the frequency rises, so does the second component’s contribution to overall conductivity [103].

## 4. Conclusions

The solid polymer electrolyte that conducts potassium ions has been made using the solution cast method at room temperature. Investigations have been conducted into how variations in salt concentration affect the structural, dielectric, and ion transport characteristics of produced films. According to the FTIR analysis, the complexation between the polymer and salt was confirmed. When KSCN salt content increased up to 40 wt.%, the free ion is dominant based on FTIR deconvolution. From the fitted EIS plot and EEC diagram, the entire figure of the system was visualized. The highest conductivity of 1.54 × 10^−7^ S cm^−1^ is achieved when the salt concentration has reached 30 wt.% based on EIS approach. The extracted ion transport parameters from the deconvoluted anion peak revealed a clear enhancement in the transport parameters for the MCK4 sample, which is not in accordance with the EIS investigations. Dielectric polarization is the explanation for the dispersion with a high value of dielectric constant in the low frequency range. The highest conducting sample has the greatest value of dielectric constant, and often follows the same pattern as the conductivity study. No discernible dielectric loss peak is seen because the electrode polarization effect might have covered it. In the *M*″ versus frequency plot, the long-range conductivity relaxation is shown as a resonance peak. The faster ion migration via the segmental motion of the polymer chain and the presence of more free charge carriers in the system may account for the improvement in dielectric properties. When the frequency-dependent conductivity shows a large flat area, it means that ions are moving, which gives rise to the conductivity.

## Figures and Tables

**Table 1:** Bulk resistance and circuit elements for MC:KSCN SPE films.

Sample	*p*1 (Rad)	*p*2 (Rad)	CPE1 (F)	CPE2 (F)	*R_b_* (Ω)	Conductivity (S/cm)
MCKN1	0.91	-	4.00 × 10^−10^	-	1.15 × 10^7^	1.34 × 10^−9^
MCKN2	0.86	-	7.41 × 10^−10^	-	1.66 × 10^6^	9.30 × 10^−9^
MCKN3	0.83	0.51	1.82 × 10^−9^	2.22 × 10^−6^	5.15 × 10^4^	2.99 × 10^−7^
MCKN4	0.86	0.43	1.33 × 10^−9^	1.43 × 10^−6^	1.00 × 10^5^	1.54 × 10^−7^
MCKN5	0.90	0.43	1.17 × 10^−9^	5.26 × 10^−7^	2.60 × 10^5^	5.93 × 10^−8^

**Table 2:** The values of *D*, *µ*, and *n* at room temperature from EIS measurement.

Sample	*µ* (cm^2^ V^−1^ s)	*D* (cm^2^ s^−1^)	*n* (cm^−3^)
MCKN1	-	-	-
MCKN2	-	-	-
MCKN3	5.92 × 10^−10^	1.52 × 10^−11^	3.16 × 10^21^
MCKN4	1.13 × 10^−9^	2.90 × 10^−11^	8.52 × 10^20^
MCKN5	3.45 × 10^−9^	8.84 × 10^−11^	1.07 × 10^20^

**Table 3:** The values of *D*, *µ*, and *n* at room temperature using FTIR method.

Sample	*n* (cm^−3^)	*µ* (cm^2^ V^−1^ s)	*D* (cm^2^ s^−1^)
MCKN1	1.94 × 10^21^	4.32 × 10^−12^	1.13 × 10^−13^
MCKN2	4.78 × 10^21^	1.21 × 10^−11^	3.17 × 10^−13^
MCKN3	1.33 × 10^22^	1.41 × 10^−10^	3.68 × 10^−12^
MCKN4	3.92 × 10^22^	2.46 × 10^−11^	6.41 × 10^−13^
MCKN5	2.15 × 10^22^	1.72 × 10^−11^	4.49 × 10^−13^
